# Outcomes of Catheter-Directed Thrombolysis for Arteriovenous Fistula Thrombosis in Singapore: Is It Still Relevant Today?

**DOI:** 10.3400/avd.oa.20-00112

**Published:** 2021-03-25

**Authors:** Clarice Biru Yeo, Enming Yong, Qiantai Hong, Justin Kwan, Lawrence Han Hwee Quek, Uei Pua, Sundeep Punamiya, Sadhana Chandrasekar, Glenn Wei Leong Tan, Zhiwen Joseph Lo

**Affiliations:** 1 Lee Kong Chian School of Medicine, Nanyang Technological University, Singapore; 2 Vascular and Endovascular Surgery, Department of General Surgery, Tan Tock Seng Hospital, Singapore; 3 Vascular and Interventional Radiology, Department of Diagnostic Radiology, Tan Tock Seng Hospital, Singapore

**Keywords:** arteriovenous fistula, thrombolysis, vascular access, dialysis

## Abstract

**Objective**: To review the outcomes of catheter-directed thrombolysis (CDT) for salvage of thrombosed arteriovenous fistula (AVF) in a single centre in Southeast Asia.

**Methods**: A retrospective study of CDT in AVF between January 2015 and July 2018 at a tertiary university hospital was carried out.

**Results**: Within the study period, 85 patients underwent CDT for AVF thrombosis. Of these patients, 78% underwent CDT for 24 h and 12% required CDT for 48 h. Moreover, 14% of patients had bleeding during CDT and hence required a decrease in dosing or complete cessation. Incidence of intracranial haemorrhage was 1%, and technical success was 92%. Post CDT, primary patency rates at 12, 24 and 36 months were 87%, 62% and 36%, respectively; assisted primary patency rates at 12, 24 and 36 months were 96%, 82% and 69%, respectively; and secondary patency rates at 12, 24 and 36 months were 99%, 93% and 86%, respectively. Multivariate analysis did not identify any predictive factors for patency post CDT.

**Conclusion**: Within our study population, CDT for AVF salvage conferred good technical results with low rates of complications. There was good primary patency at 12 months, and the results were sustained up to 36 months. It remains a useful modality for fistula salvage, avoiding surgical intervention.

## Introduction

The Global Burden of Disease study estimated that 1.2 million people died from end-stage renal failure (ESRF) in 2015, an increase of 32% since 2005.^1)^ Singapore currently ranks fourth in the world for prevalence of ESRF. These numbers continue to grow, with Singapore ranking sixth globally for newly diagnosed cases of renal failure and a new dialysis patient gained approximately every 5 h.^2)^ These statistics indicate the importance of addressing the healthcare needs of ESRF patients, with arteriovenous fistula (AVF) patency being of utmost importance as AVFs are currently recommended as the first choice for haemodialysis access.^3,4)^ Late complications of AVF include infection, thrombosis, distal ischemia, venous oedema and cardiac failure. AVF thrombosis is a serious complication and is the leading cause of permanent access loss.^5)^ Patent vascular access is a prerequisite for adequate haemodialysis and is a major determinant of quality of life and long-term survival in an ESRF patient.^6)^ With a limited number of possible access sites, AVF thrombosis necessitates definitive treatment of the clinically significant culprit lesion to ensure optimal vascular access.^3)^

Endovascular approaches have become the first line of treatment for AVF thrombosis, as it is less invasive than open surgery. The surgical approach is being reserved for endovascular treatment failures. One of the benefits of endovascular approaches is the preservation of the original anastomosis and conduit. In contrast, surgical salvage may require redo anastomosis, creation of a new fistula higher in the arm (e.g., proximal forearm) or insertion of a graft. The reduced need for surgical reinterventions and new fistulas seems to be a direct consequence of this strategy.^7)^ Although there is significant literature available on catheter-directed thrombolysis (CDT) and its effectiveness in the salvage of thrombosed AVFs, there are limited studies that involve Southeast Asian populations.^7–10)^ Hence, this study aims to review the outcomes of CDT in AVF salvage within a single centre in Southeast Asia, to verify its effectiveness in treatment and viability as an alternative to traditional surgical methods.

## Methods

### Patients and hospital setting

A total of 85 patients with AVF thrombosis underwent CDT between January 2015 and July 2018. Ethics approval for the study was obtained from our hospital’s Institutional Review Board (NHG DSRB Ref: 2020/00752). All AVF CDT procedures and subsequent fistuloplasties were performed by consultant grade interventional radiologists in a 1500-bed tertiary care university hospital in central Singapore. An endovascular-first approach was followed. The inclusion criterion was patients who presented with thrombosed AVF. Patients who were older than 80 years and with contraindications to thrombolysis were excluded. These contraindications include allergy to recombinant tissue plasminogen activator (rTPA), bleeding diathesis, recent gastrointestinal bleeding, significant cerebrovascular accident including haemorrhage or large infarction within the previous 6 months or recent abdominal surgery within the previous 3 months.

### Procedure

The AVF was accessed antegrade or retrograde under ultrasound-guided percutaneous puncture, and 6Fr sheath inserted. A multi-level infusion catheter was then placed under fluoroscopy guidance following crossing of the thrombosed AVF with hydrophilic wire. The catheters used were either Fountain thrombolysis infusion catheters (Merit Medical, Galway, Ireland) or Cragg-McNamara catheters (ev3 Inc., Plymouth, MN, USA). The sheaths were secured to the skin by a stitch, and the CDT catheters were additionally secured to the catheters to prevent dislodgement. These were secured with light gauze and dressing.

An initial pulsed dose of 15 mg rTPA (alteplase) was given and subsequent 24- to 48-h infusion of 0.5 mg/h of rTPA administered via the catheter in high dependency as per a lyse and wait strategy, as first described by Cynamon in 1997.^11)^ Patients were kept under observation in the high-dependency unit with hourly parameters; their sheath site and Glasgow Coma Score were monitored. A saline infusion was given at 12 ml/h via the side port of the 6Fr sheath to maintain sheath patency, and additional intravenous heparin of 200 IU/h was also given. A check angiogram was routinely performed the following day, and if clot burden was significantly reduced, balloon maceration and fistuloplasty were performed to treat the culprit lesion. CDT would be continued if clot burden was still heavy but not beyond 48 h. All patients had angioplasty to the culprit lesion following CDT. Covered stenting was reserved for patients with rupture that did not resolve with prolonged low-pressure balloon tamponade.

We adapted the definitions laid down by the Committee on Reporting Standards of the Society for Vascular Surgery and the American Association for Vascular Surgery for the purpose of this study.^12)^

In this study, we have also defined failure of thrombolysis as either the recurrence of AVF thrombosis in the same segment or AVF failure within a duration of 6 months.

### Statistical analysis

Statistical analysis was conducted using Microsoft Excel 2016 and Real Statistics Resource Pack for Excel 2016. Descriptive analysis was conducted for all patient demographical data. To evaluate the influence of patient demographics on the outcome of CDT, univariate analysis was done by performing a t-test on continuous data and a Fisher exact test on categorical data. A p-value of <0.05 was considered statistically significant. Reintervention-free survival was illustrated with Kaplan–Meier curves.

## Results

The cohort of 85 patients with thrombosed AVFs underwent CDT from 2 January 2015 to 11 July 2018. **Table 1** shows the demographic data for the patients analysed in this study. Within the study population of patients with thrombosed AVFs, 60% were male, average age was 61 years and 74% of patients have their AVFs created on the left arm. The ethnicity make-up of the study population, with 69% Chinese, 19% Malay and 8% Indian, was similar to that of the general Singapore population.^13)^ The majority of patients (73%) was on an antiplatelet regime.

**Table 1:** Table 1 Study population characteristics

**Demographics**	
Study population	85
Study period	2 January 2015 to 11 July 2018
Male : female	51 (60%) : 34 (40%)
Chinese : Malay : Indian : Others	59 (69%) : 16 (19%) : 7 (8%) : 3 (4%)
Mean age (range)	61 (31–82) years
**AVF characteristics**	
Right AVF : left AVF	22 (26%) : 63 (74%)
Brachiocephalic (BC) : radiocephalic (RC) AVF : brachiobasilic (BB)	52 (61%) : 20 (24%) : 13 (15%)
Previous fistuloplasty	47 (55%)
Previous CDT	10 (12%)
Mean AVF age	31 months
Femoral catheter inserted	45 (53%)
**Medications**	
Aspirin	41 (48%)
Plavix	21 (25%)
Other anticoagulants	1 (1%)

AVF: arteriovenous fistula; CDT: catheter-directed thrombolysis

Of the thrombosed AVFs in our study, 61% were brachiocephalic AVFs, 24% were radiocephalic AVFs and 15% were brachiobasilic AVFs. Seventy-four per cent of AVFs were located over the patient’s left arm, whereas 26% of AVFs were located over the patient’s right arm.

Of the patients, 43% had thrombosis originating from culprit lesions, the juxta-anastomosis; another 43% had thrombosis originating from the venous limb; and the remaining 14% had thrombosis arising from the cephalic arch.

A technical success rate of 92% was achieved in 78 out of 85 cases (**Table 2**). They all had reliable and successful cannulation after re-establishment of vascular access patency. Technical failures accounted for seven out of 85 cases (8%). The majority of the technical failures (four out of seven cases) was attributed to high clot burden resulting in failure of thrombolysis. The other three were due to rupture following fistuloplasty secondary to underlying vessel disease, that was not amenable to further prolonged balloon tamponade or stenting. Of the endovascular technical failures, one AVF was subsequently successfully salvaged with open surgical thrombectomy, whereas the rest of the accesses were abandoned.

**Table 2:** Table 2 CDT procedure outcomes

Success	78 (92%)
Failure	7 (8%)
Average length of stay (range)	12 (3–89) days
**CDT duration**	
24 h	75 (88%)
48 h	10 (12%)
**Complications**	
Bleeding	12 (14%)
Intracranial haemorrhage	1 (1%)
30-day mortality	3 (3%)
**Site of AVF thrombosis**	
Cephalic arch	12 (14%)
Juxta-anastomosis	37 (43%)
Venous limb	37 (43%)

AVF: arteriovenous fistula; CDT: catheter-directed thrombolysis

Throughout the duration of this study, 14% of patients developed complications in the form of minor bleeding. Minor bleeding was defined as bleeding not requiring surgical intervention or transfusions. Blood was usually oozing from the CDT sheath insertion site or from the patients’ temporary dialysis catheter insertion site. Only one patient suffered from major bleeding complications. This patient (1%) sustained a major bleed with an intracranial haemorrhage, which required surgical intervention. The patient was ultimately discharged with poor neurological prognosis. Among patients with bleeding complications (14% of the study population), there was no significant difference between aspirin and clopidogrel utilisation. Univariate analysis did not show any statistically significant factors among AVF characteristics and intraoperative findings on outcome.

Technical success was achieved in all 10 patients who required extended 48-h CDT. Of the 10 patients, one had local bleeding from the sheath that was self-limiting. None of the patients in this group who underwent extended CDT suffered from complications such as intracranial haemorrhage or death within 30 days.

The 30-day mortality was 3.5%. The causes of death were intracranial haemorrhage (same case as above) and unrelating aetiologies such as myocardial infarction and spontaneous bacterial peritonitis secondary to ascites. The average and median length of inpatient stay was 12 and 10 days, respectively, with one outlier who stayed for 89 days. This patient had concomitant critical limb ischaemia with attempts at limb salvage requiring foot debridement and amputation.

## Discussion

The mechanism of AVF thrombosis is proposed to be due to progressive stenosis, usually arising from the arteriovenous anastomosis. Methods of treatment include surgical (open thrombectomy±reanastomosis or interposition graft placement), endovascular or hybrid procedures. Meta-analyses have shown similar technical success rates of endovascular treatment of thrombosed fistula (73–96%) and surgical therapy (66–95%).^14)^ There are a myriad of endovascular devices available, although pharmacomechanical thrombolysis and endovascular mechanical thrombectomy alone have both been shown to yield similar outcomes.^14)^ Endovascular approaches offer distinct advantages over surgical approaches, including lower complication rates. Surgical approaches tend to be associated with more serious complications such as infection, as well as risks of anaesthesia.^15,16)^ This has resulted in endovascular approaches largely replacing surgical approaches and thus being recommended as the first line of treatment in thrombosed AVFs today.^4)^

**Figure 1:**
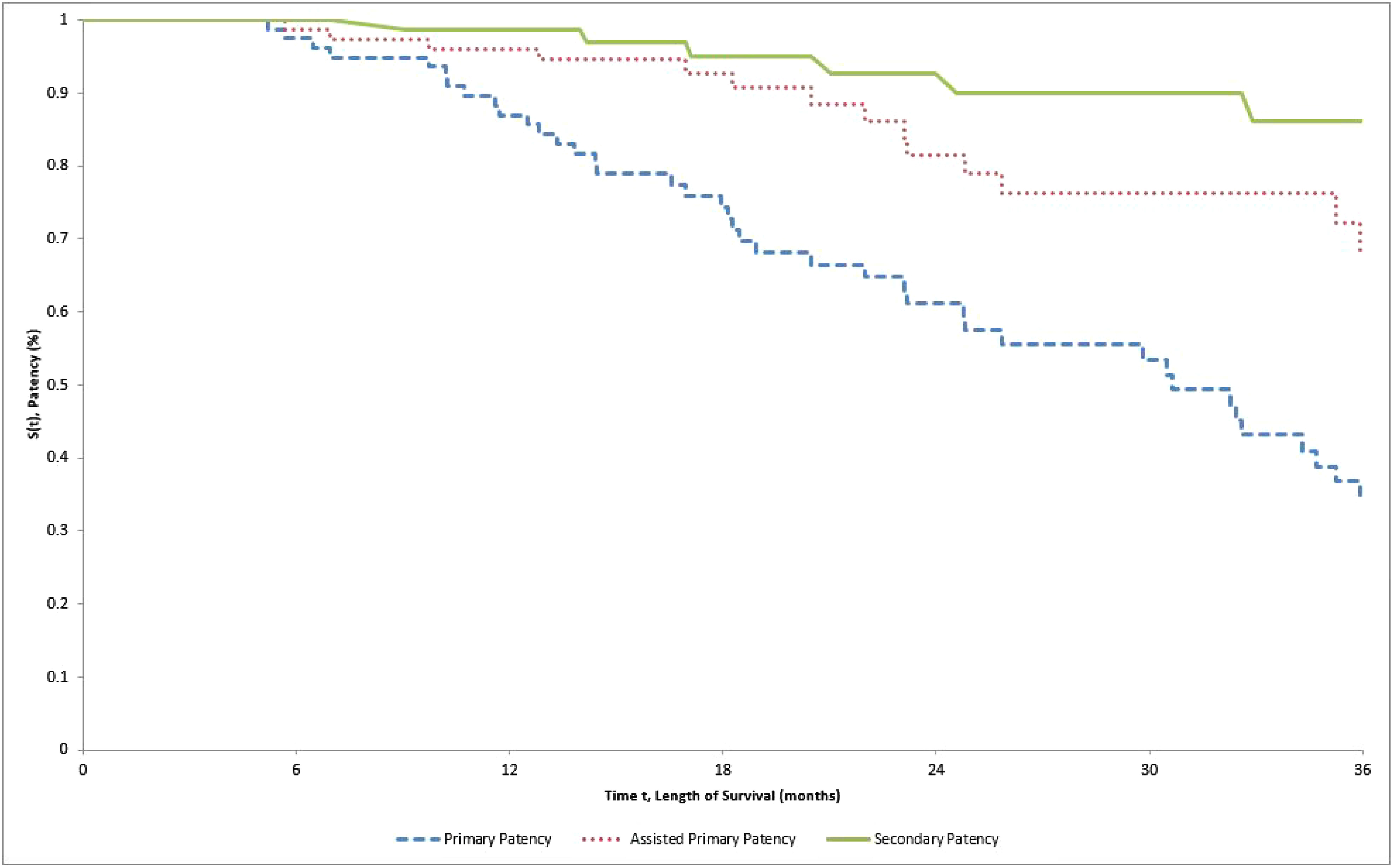
Fig. 1 Kaplan–Meier curve for patency rates for 24- and 48-h CDT.

We have achieved a technical success rate of 92% (**Table 2**), which is consistent with the rates (90% or better) reported in the National Kidney Foundation Dialysis Outcomes Quality Initiative (NKF KDOQI) guidelines for stenosed and occluded AVFs.^4)^ Post CDT, our primary patency rates at 12, 24 and 36 months were 87%, 62% and 36%, respectively; assisted primary patency rates at 12, 24 and 36 months were 96%, 82% and 69%, respectively; and our secondary patency rates at 12, 24 and 36 months were 99%, 93% and 86%, respectively (**Fig. 1**). These results exceed the 2006 NKF KDOQI guidelines for stenosed and occluded AVFs (50% primary patency rate at 6 months). In the 10 patients who underwent extended 48-h CDT, primary patency was also maintained at 88% at 6 and 12 months, with low morbidity (one minor bleeding) and no deaths, thus showing that fistula salvage is still possible in this group, that the majority of thrombosed AVFs can be salvaged by CDT and that AVF patency at short- to medium-term intervals is still preserved post CDT. CDT is effective in prolonging the lifespan of AVFs for haemodialysis access, and we were able to postpone the need for surgical intervention and the creation of a new AVF.

Our technical success rates, as well as post-CDT primary and secondary patency rates, at 12 and 24 months are comparable with those achieved in studies done in different populations. Technical success rates grossly range from 88% to 98% in the literature, whereas post-CDT primary patency rates at 12 and 24 months range from 42% to 68% and 52% to 67%, respectively, and post-CDT secondary patency rates at 12 and 24 months range from 68% to 72%.^8–12)^ This therefore highlights the finding that the results for CDT for AVF salvage can be extended to a Southeast Asian population.

Complications encountered when performing CDT include bleeding and can lead to further complications such as extended hospital stay, surgery, transfusion and death.^17)^ In our study, 12 patients (14%) experienced complications in the form of local bleeding (**Table 2**). In resolving these bleeds, the thrombolytic agents used were either reduced in dose or stopped. One patient unfortunately suffered from an intracranial haemorrhage with fatal consequences (**Table 2**). Bleeding complications are attributed to the use of thrombolytic agents such as rTPA or anticoagulation such as heparin, which can cause an increased bleeding risk. No single thrombolytic agent is immune to the risk of bleeding.^16)^ Contraindications for CDT include allergy to rTPA, bleeding diathesis, recent gastrointestinal bleeding, significant cerebrovascular accident including haemorrhage or large infarction within the previous 6 months or surgery within the previous 3 months.^18)^ In addition to the factors listed, our centre uses a cut-off age of 80 years to decide whether patients are candidates for CDT.

The use of fibrinogen assay as a surrogate to guide rTPA dosing has been described to monitor thrombolysis efficacy and risk of bleeding complications. A survey of interventional radiologists from the Society of Interventional Radiology showed 82% were reporting its use during thrombolysis. The literature, however, is still inconclusive, with studies showing both increased and reduced risk of bleeding at fibrinogen thresholds of less than 150 mg/dl.^19)^ A meta-analysis of its use in arterial CDT revealed varying protocols for fibrinogen measurement and that its role for predicting haemorrhagic complications after CDT remains unproven because of a limited number of heterogenous studies.^20)^

There are other modalities for endovascular thrombus removal besides CDT. These include pulse-spray thrombolysis, which is an evolution of the traditional lyse and wait technique. This technique can be performed with or without thrombo-aspiration using a catheter or clot maceration using a balloon catheter (pharmacomechanical thrombectomy). However, a retrospective study on 68 episodes of graft occlusion in New Jersey using the pulse-spray thrombolysis technique with the aid of an over-the-wire Fogarty balloon for clot removal only showed a primary patency rate of 44% and a secondary patency rate of 72% at 180 days. Although the procedural success rate was 95% with a mean rTPA dose of 4 mg, the patency rates were inferior to those of CDT.^21)^ Choi et al. also reported good results using pharmacomechanical thrombectomy through a combination of the pulse-spray technique with urokinase followed by thrombus maceration with a balloon catheter in thrombosed arteriovenous grafts. This showed a technical and clinical success rate of 95% although post-intervention primary patency rates were 45% and 22% at 12 and 24 months and secondary patency rates were 96% and 91% at 12 and 24 months, respectively.^22)^

Additional modalities include mechanical thrombectomy devices (Cleaner™ Rotational Thrombectomy System, Rex Medical, Athens, TX, USA), pharmacomechanical devices (Angiojet® Rheolytic thrombectomy catheter, Boston Scientific, Marlborough, MA, USA) and suction thrombectomy devices (Indigo® Thrombectomy Aspiration System, Penumbra, Alameda, CA, USA).^23)^ Following the availability of mechanical thrombectomy devices in our institution, we have been using such devices as a one-stage procedure, coupled with bolus thrombolysis of rTPA delivered via the Berman catheter, with the number of CDT procedures correspondingly falling. One-stage procedures reduce the risk of troublesome bleeding and avoid the need for high-dependency monitoring while resulting in a reduction of overall length of stay. Patient selection is still required as such devices may have difficulty removing clot in aneurysmal segments of AVF, and we have experienced technical and clinical failure following use of such devices, sometimes necessitating reintervention with CDT.

Our institution experience is also consistent with the change of declotting procedures from thrombolysis-dependent to thrombectomy-dependent procedures globally, with the advent of new thrombectomy devices. A recent meta-analysis reviewed 17 studies with 3000 cases of percutaneous treatment of thrombosed vascular access. It showed no significant difference between thrombolysis and thrombectomy-dependent procedures with regard to dialysis access circuit survival. A trend of improvement in assisted primary patency rates over time was, however, demonstrated as studies after 2009 showed better dialysis circuit survival rates. This was explained by improvement in devices, techniques and the use of drug-coated balloons, covered stents and cutting balloons that have also helped in improving patency rates of dialysis circuits.^24)^ More relevant for our study is the fact this review also showed that the lyse and wait strategy had one of the highest post-intervention patency rates and that thrombectomy procedures using dedicated devices failed to significantly improve assisted primary patency rates, although the latter had additional benefits of lower usage of lytic medications to reduce complications.

## Strengths and Limitations

To our knowledge, this is the first paper to describe results of CDT in a Southeast Asian population. Limitations include the retrospective nature of our review. It is also a single-institution study with a small sample size, so selection bias may exist. Lastly, because of institutional practice, the CDT was performed over 24 to 48 h, resulting in increased length of stay. We are now moving towards one-stage procedures with endovascular thrombus removal.

## Conclusion

CDT in thrombosed AVFs is safe. Within our study population of Southeast Asian ethnicity and mean AVF age of 34 months, CDT for AVF salvage conferred good technical results with low rates of complications. There was a primary patency rate of 87% at 12 months following fistula salvage. The results were sustained up to 36 months, with a primary patency rate of 69% and a secondary patency rate of 86%. It remains a useful modality for fistula salvage, avoiding surgical intervention. This helps to delay access abandonment which will result in the need to create a new surgical AVF. The use of newer techniques and novel dedicated thrombectomy devices show promise, but patient selection is important.
