# Supplementary material for: Outcomes of Catheter-Directed Thrombolysis for Arteriovenous Fistula Thrombosis in Singapore: Is It Still Relevant Today?
Source: Ann Vasc Dis. 2021 Mar 25;14(1):5–10. doi: 10.3400/avd.oa.20-00112 (PMC7991696; doi:10.3400/avd.oa.20-00112)
Supplement: Supplementary Data [file avd-14-1-oa.20-00112_s001.pdf]

Supplementary Figures

Supplementary Figure 1

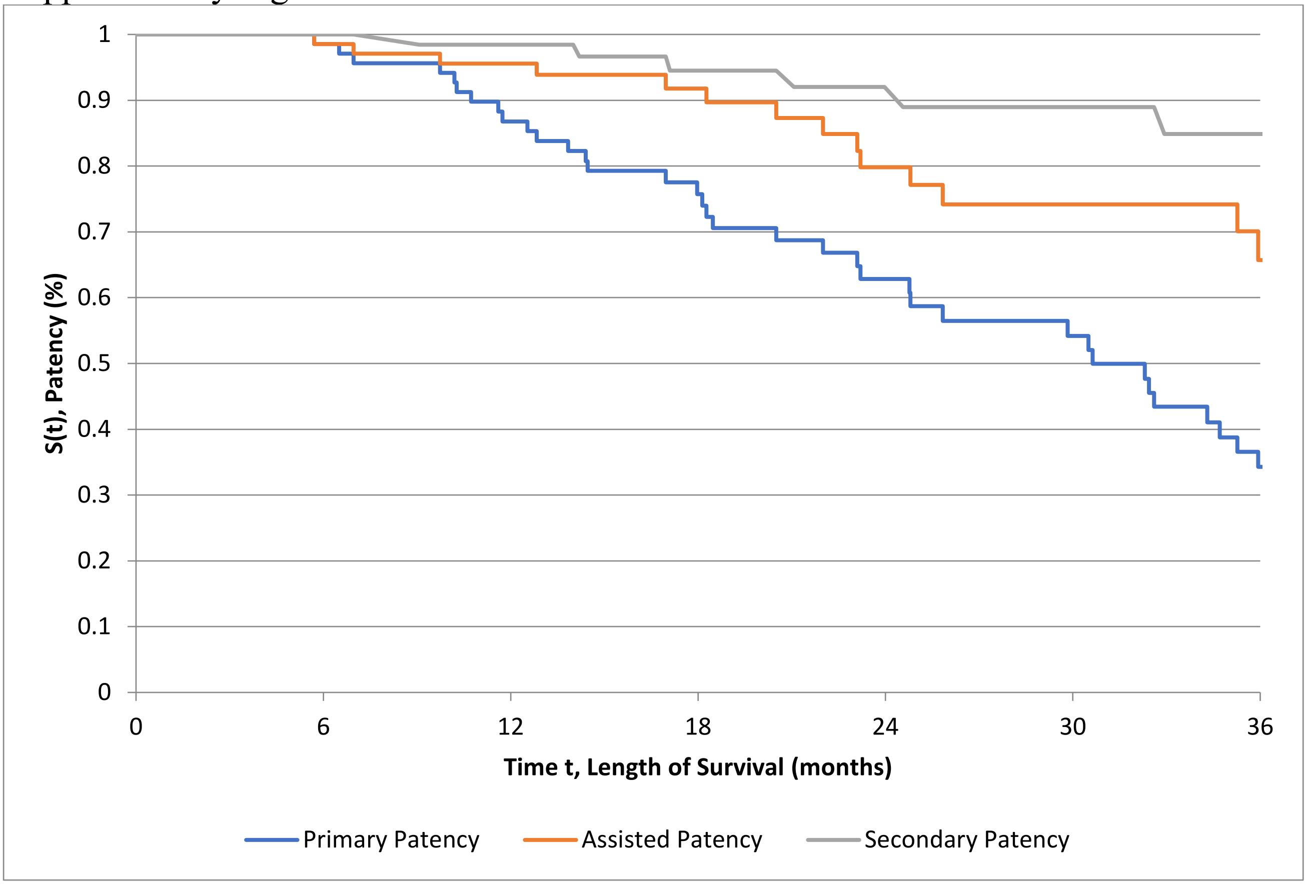

Supplementary Figure 2

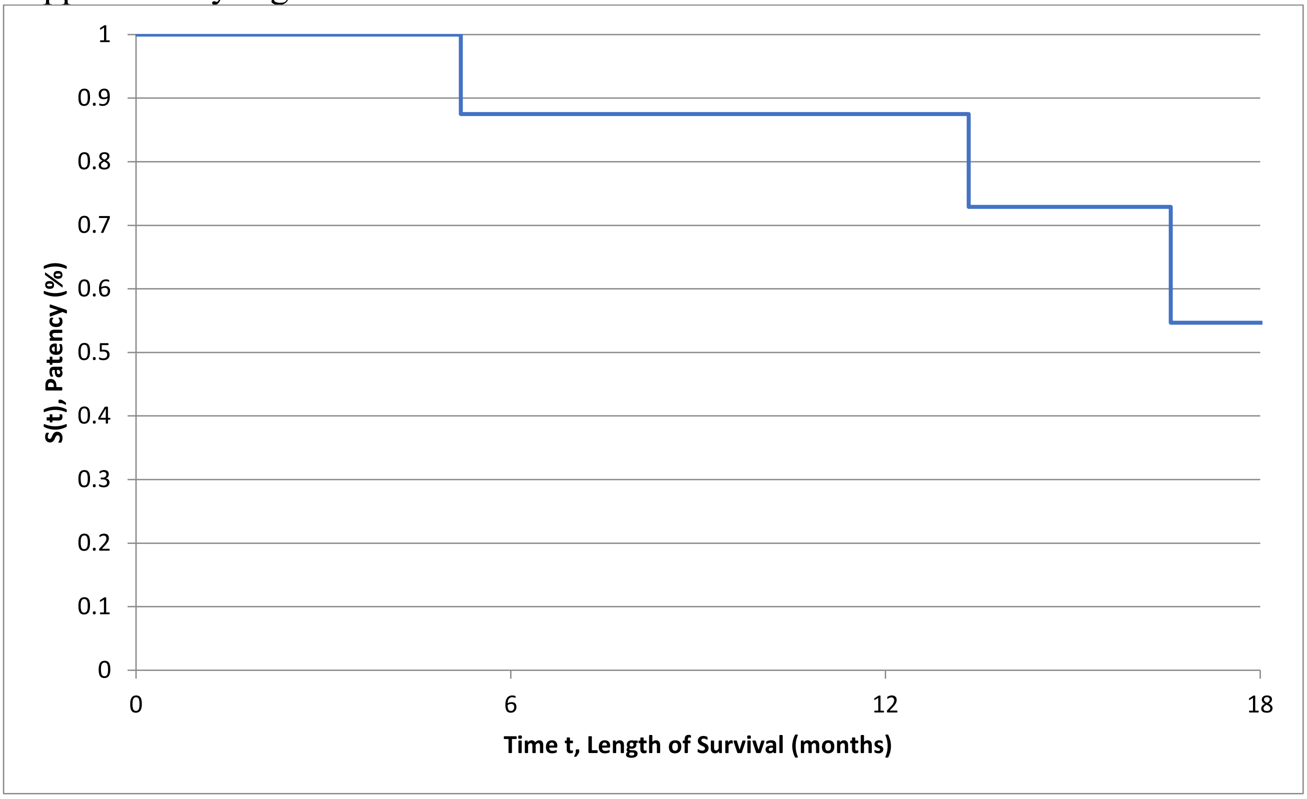

Supplementary Figure 3

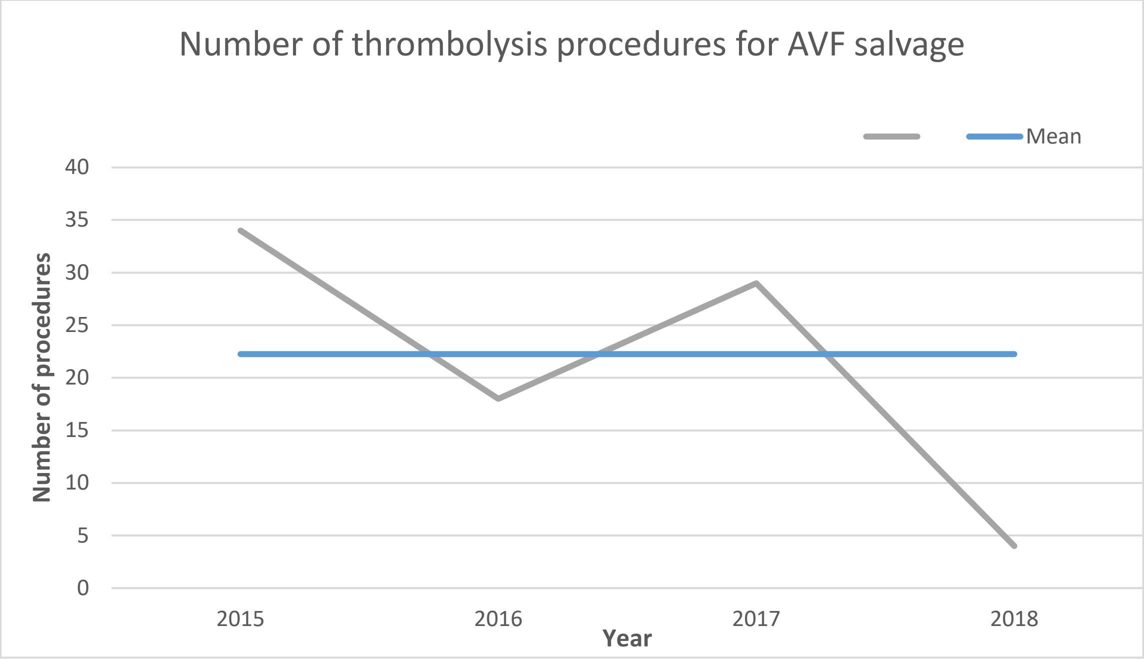

Supplementary Figure legends

Supplementary Figure 1: Kaplan–Meier survival curve for 24 hr lysis time

Number at risk							
Primary patency	75	74	66	59	52	48	39
Assisted primary patency	75	74	72	70	65	63	61
Secondary patency	75	75	74	72	71	70	69

Supplementary Figure 2: Kaplan–Meier survival curve for 4 hr lysis time—primary patency

Number at risk				
Primary patency	10	9	7	5

Supplementary Figure 3: Number of thrombolysis procedures for AVF salvage
